# Chinese calligraphy as cultural mediation: a cultural-historical activity theory perspective on therapeutic practice for neuropsychiatric symptoms

**DOI:** 10.3389/fpsyt.2025.1686995

**Published:** 2025-10-16

**Authors:** Kuan-Yu Chu

**Affiliations:** ^1^ School of Dentistry and Graduated Institute of Dental Science, College of Oral Medicine, National Defense Medical University, Taipei, Taiwan; ^2^ Interdisciplinary Education Center, MacKay Junior College of Medicine, Nursing and Management, Taipei, Taiwan

**Keywords:** mental health, cross-cultural comparison, psychotic disorders, mental health services, community participation

## Abstract

Chinese calligraphy therapy (CCT) represents an emerging culturally mediated intervention demonstrating significant therapeutic potential for neuropsychiatric symptoms including anxiety, depression, schizophrenia, and cognitive impairment. This perspective integrates Cultural-Historical Activity Theory (CHAT) to elucidate the psychosocial mechanisms underlying CCT’s effectiveness, synthesizing meta-analytic evidence that demonstrates standardized mean differences of -0.17 for psychosis reduction and significant improvements in anxiety symptoms. The framework reveals how traditional calligraphic practice functions as a mediational tool, facilitating psychological transformation through cultural mediation, community participation, and zone of proximal development activation. Current research indicates CCT’s capacity to enhance neural efficiency, promote flow states, and improve cognitive function in diverse populations. This perspective advocates for systematic integration of culturally authentic practices within evidence-based mental healthcare, proposing future directions including longitudinal studies, cross-cultural validation, and community-based implementation. The analysis contributes to understanding how traditional therapeutic modalities can address contemporary mental health challenges through theoretically grounded, culturally responsive approaches.

## Introduction

The increasing prevalence of neuropsychiatric symptoms globally necessitates innovative therapeutic approaches that transcend conventional Western treatment paradigms. Chinese calligraphy therapy (CCT) has emerged as a promising culturally mediated intervention, demonstrating measurable effects on anxiety, depression, psychosis, and cognitive impairment across diverse clinical populations (**Table 1**). Recent meta-analytic evidence indicates CCT significantly reduces psychosis symptoms (standardized mean difference -0.17, 95% CI [-0.40, -0.30], p < 0.01) and anxiety manifestations across 21 studies encompassing 965 participants (1).

**Table 1:** Summary of key studies examining chinese calligraphy therapy for neuropsychiatric symptoms.

Study	Design	Population	Sample size	Intervention duration	Control group	Primary outcome measures	Key outcomes	Effect size
Chu et al., 2018 (1)	Meta-analysis	Neuropsychiatric symptoms	965 (21 RCTs)	4–24 weeks (mean: 12 weeks)	Usual care, waitlist, attention control	PANSS, HARS, HAMD, cognitive assessments	Anxiety SMD -0.78, depression SMD -0.69, psychosis reduction	SMD -0.78 anxiety, -0.69 depression
Hsiao et al., 2023 (2)	Exploratory	Mild cognitive impairment	30	8 weeks	No intervention control	Self-report questionnaires, cognitive tests	Cognitive and emotional improvements	Self-reported benefits
Liu et al., 2023 (3)	Network meta-analysis	Dementia	2,801 (39 RCTs)	Variable (2–52 weeks)	Usual care, other art therapies	MMSE, ADL, QoL scales	CCT most effective art therapy	MD 4.39 cognition, 9.00 QoL vs usual care
Huang et al., 2022 (4)	RCT	Schizophrenia	150	24 weeks	Standard psychiatric care	PANSS, cognitive battery, functional assessment	Cognition increase, symptom decrease	Moderate effects
Chan et al., 2017 (5)	RCT	Older adults at risk of MCI	83	16 weeks	Active control (health education)	Attention tests, working memory tasks	Improved attention and working memory	Cohen’s d = 0.52-0.68
Wang & Tang, 2024 (6)	Cross-sectional	Older Chinese adults	312	Ongoing practice (mean: 5.2 years)	Non-practitioners	Peace of mind scale, stress management, health perception	Significant correlations with well-being	r = 0.34-0.58

RCT, Randomized Controlled Trial; SMD, Standardized Mean Difference; MD, Mean Difference; PANSS, Positive and Negative Syndrome Scale; HARS, Hamilton Anxiety Rating Scale; HAMD, Hamilton Depression Rating Scale; MMSE, Mini-Mental State Examination; ADL, Activities of Daily Living; QoL, Quality of Life; MCI, Mild Cognitive Impairment.

The theoretical foundation for understanding CCT’s therapeutic mechanisms requires frameworks that account for cultural mediation, social interaction, and developmental processes. Cultural-Historical Activity Theory (CHAT) provides a comprehensive lens for analyzing how traditional practices function as psychological tools (7), mediating between individual cognition and sociocultural contexts. This perspective examines current advances in CCT research through CHAT principles, elucidating mechanisms of therapeutic action and proposing future directions for research and clinical implementation.

Contemporary mental health research increasingly recognizes the limitations of purely biomedical approaches, calling for integration of culturally responsive interventions that address both symptom reduction and holistic well-being. CCT represents a paradigmatic example of how traditional practices can be systematically integrated into evidence-based mental healthcare, offering culturally authentic alternatives that resonate with specific populations while demonstrating measurable clinical outcomes (8).

## Cultural-historical activity theory framework

CHAT conceptualizes human psychological development as mediated through cultural tools and social interaction within specific activity systems (9). The framework comprises three interconnected levels: individual psychological processes, interpersonal relationships, and broader sociocultural contexts. In therapeutic applications, CHAT emphasizes how cultural practices function as mediational tools, facilitating transformation through guided participation and collaborative meaning-making.

The zone of proximal development (ZPD) represents a central CHAT concept, describing the space between independent performance and potential development through guided practice (10). In CCT contexts, master calligraphers or therapists scaffold participants’ skill development, creating opportunities for psychological growth that extend beyond technical proficiency to encompass emotional regulation, attention control, and self-awareness.

Cultural mediation occurs through the symbolic and material aspects of calligraphic practice. The physical act of brush manipulation, character formation, and ink flow creates embodied experiences that mediate cognitive and emotional processes. Simultaneously, the cultural meanings embedded in Chinese characters and aesthetic principles provide symbolic resources for psychological transformation.

Activity theory’s emphasis on object-oriented practice aligns with CCT’s goal-directed nature (11). Participants engage in meaningful activities that connect individual therapeutic objectives with broader cultural values and community participation. This integration creates sustainable motivation and authentic engagement that transcends clinical intervention boundaries.

**Figure 1:**
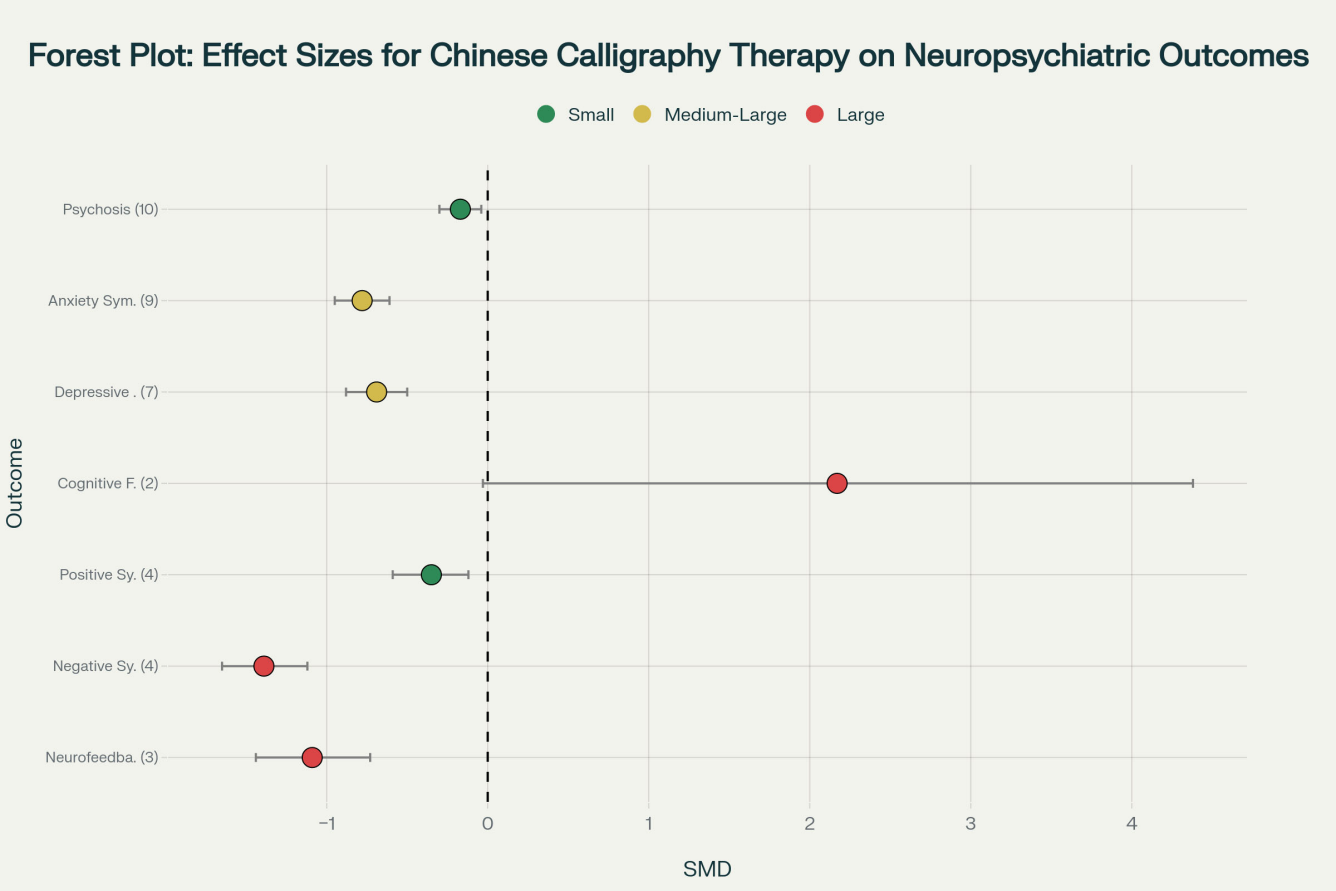
Forest plot showing standardized mean differences (SMD) and 95% confidence intervals for the effects of Chinese calligraphy therapy on neuropsychiatric outcomes. Effect sizes are categorized as small (green), medium-large (yellow), and large (red) according to Cohen's conventions. Numbers in parentheses indicate the number of studies included for each outcome. The vertical dashed line represents no effect (SMD = 0).

**Figure 1** illustrates the CHAT framework as applied to Chinese Calligraphy Therapy, showing the interconnections between mediational tools (brush, characters, cultural meanings), the zone of proximal development (scaffolded learning), and therapeutic outcomes across individual, interpersonal, and sociocultural levels.

## CHAT analysis of Chinese calligraphy therapy

### Mediational tools and psychological transformation

CCT operates through multiple mediational tools that facilitate therapeutic change. The brush functions as a physical mediator, requiring fine motor control that enhances concentration and mindfulness (12). Neuroimaging studies demonstrate that calligraphic practice activates neural networks associated with flow states, characterized by heightened focus and positive affect (13). These findings suggest that the physical act of calligraphy creates neurobiological conditions conducive to therapeutic change.

Character formation serves as symbolic mediation, connecting individual expression to cultural meaning systems. Research indicates that engagement with Chinese characters activates both motor and cognitive networks, creating integrated experiences that promote holistic well-being (2). The semantic richness of characters provides resources for emotional processing and meaning-making that extend beyond conventional verbal therapies.

### Zone of proximal development in therapeutic practice

CCT interventions typically involve structured progression from basic strokes to complex characters, creating optimal challenge levels that promote skill development without overwhelming participants (14). This scaffolding approach aligns with ZPD principles, ensuring that therapeutic activities remain within participants’ capabilities while promoting growth. Studies with schizophrenia patients demonstrate significant cognitive improvements when calligraphic activities are appropriately calibrated to individual skill levels.

The social dimension of ZPD manifests in group calligraphy sessions, where peer interaction and instructor guidance create collaborative learning environments. Research indicates that group art therapy incorporating traditional Chinese materials, including calligraphy, enhances social function and self-efficacy among individuals with schizophrenia. These findings highlight how CHAT’s emphasis on social mediation translates into measurable therapeutic outcomes (15).

### Cultural context and community participation

CCT’s therapeutic effectiveness is enhanced by its cultural authenticity and community connections (16). For Chinese populations, calligraphy practice connects individual therapeutic work to broader cultural identity and values, creating sustainable motivation for continued engagement. Studies with older Chinese adults demonstrate that calligraphy activities significantly correlate with peace of mind, stress self-management, and positive health perceptions (6).

The cultural dimension extends beyond ethnic identity to include intergenerational transmission and community practices. CCT interventions that incorporate traditional teaching methods and cultural contexts demonstrate higher adherence rates and more sustained benefits compared to culturally adapted approaches (17). This evidence supports CHAT’s emphasis on authentic cultural participation as essential for meaningful psychological development.

## Current advances

### Empirical evidence

Recent systematic reviews and meta-analyses provide robust evidence for CCT’s therapeutic effectiveness across multiple neuropsychiatric conditions. A comprehensive meta-analysis of 21 studies involving 965 participants demonstrated significant reductions in psychosis symptoms (SMD -0.17, p < 0.01) and anxiety manifestations. Subgroup analyses revealed differential effects across populations, with older adults showing particularly strong responses to calligraphic interventions (1).

Neurobiological research using functional magnetic resonance imaging reveals that calligraphic practice enhances neural efficiency and promotes flow states associated with positive affect and reduced stress responses (18–20). These studies demonstrate increased connectivity between attention networks and emotional regulation systems during calligraphic activities, providing neurobiological support for observed therapeutic benefits.

Clinical trials with specific populations yield promising results. A randomized controlled trial with schizophrenia patients demonstrated significant improvements in cognitive function and symptom severity following calligraphic interventions (4). Similarly, studies with mild cognitive impairment populations show beneficial effects on memory, attention, and behavioral regulation through structured calligraphy training (5).

Research on traditional Chinese exercises, including calligraphy, demonstrates effectiveness comparable to established interventions such as cognitive behavioral therapy for anxiety and depression treatment (21). Network meta-analyses indicate that traditional Chinese practices offer high adherence rates, low costs, and cultural relevance that enhance treatment engagement and sustainability (3).

Cross-cultural validation studies are emerging, examining CCT’s effectiveness in non-Chinese populations (22). Preliminary findings suggest that the therapeutic mechanisms of concentration enhancement, motor skill development, and aesthetic engagement transcend cultural boundaries, though effect sizes may vary based on cultural familiarity and meaning attribution (23).

## Discussion

The integration of CHAT with CCT research reveals several key insights for therapeutic practice and future research directions. First, the framework demonstrates how traditional practices can be understood through contemporary psychological theory without losing their cultural authenticity. CHAT’s emphasis on mediation, development, and cultural context provides a theoretical foundation for understanding why CCT demonstrates measurable therapeutic effects across diverse populations and conditions (24).

Second, the analysis highlights the importance of cultural mediation in therapeutic interventions (25). CCT’s effectiveness appears to derive not only from its technical components but from its embeddedness in meaningful cultural practices that connect individual therapeutic work to broader identity and community participation. This finding has implications for developing culturally responsive mental health services that go beyond surface-level cultural adaptations to incorporate authentic cultural practices.

Third, the research suggests that CCT’s therapeutic mechanisms operate through multiple pathways simultaneously. Neurobiological effects on attention and emotional regulation combine with psychological benefits of skill development, cultural connection, and social participation (26). This multi-modal approach may explain CCT’s effectiveness across diverse neuropsychiatric conditions and populations.

### Future directions

Several research priorities emerge from this analysis. Longitudinal studies are needed to examine CCT’s sustained effects and optimal dosing parameters. Current research primarily focuses on short-term interventions, limiting understanding of long-term therapeutic benefits and maintenance strategies. Extended follow-up studies would inform clinical protocols and help establish CCT as a sustainable therapeutic modality.

Cross-cultural validation studies should expand beyond Chinese populations to examine CCT’s effectiveness in diverse cultural contexts. Such research would help distinguish between culture-specific and universal therapeutic mechanisms, informing broader applications while respecting cultural authenticity. Comparative studies with other traditional practices from different cultures could illuminate common therapeutic principles.

Mechanism-focused research should continue investigating the neurobiological and psychological processes underlying CCT’s effectiveness. Advanced neuroimaging techniques, combined with detailed behavioral assessments, could elucidate how calligraphic practice influences neural networks associated with attention, emotion regulation, and cognitive function. This research would support evidence-based protocol development and personalized intervention approaches.

Implementation research is crucial for translating research findings into clinical practice. Studies examining optimal training requirements for therapists, cost-effectiveness analyses, and integration with existing mental health services would support widespread adoption. Community-based implementation studies could examine how CCT interventions function in naturalistic settings and identify factors that promote successful program sustainability.

Technology integration represents an emerging frontier, with digital platforms potentially expanding CCT accessibility while maintaining therapeutic authenticity. Research on virtual reality calligraphy environments, tablet-based practice systems, and telehealth delivery models could address geographical and resource barriers while preserving essential therapeutic components (27).

### Clinical implications

The evidence supports incorporating CCT into comprehensive mental health treatment approaches, particularly for populations with cultural connections to Chinese traditions. Clinical protocols should emphasize proper instructor training, appropriate progression structures, and integration with conventional treatments. The research suggests CCT functions effectively as both primary intervention for mild to moderate symptoms and adjunctive treatment for severe mental health conditions.

Healthcare systems should consider CCT’s cost-effectiveness and high adherence rates when evaluating therapeutic options. The intervention’s minimal equipment requirements and group delivery potential make it accessible for resource-limited settings while maintaining therapeutic integrity. Training mental health professionals in CCT delivery could expand treatment options and improve cultural responsiveness.

### Limitations and considerations

Current research limitations include heterogeneity in intervention protocols, outcome measures, and population characteristics across studies. Standardization of CCT delivery methods and assessment tools would strengthen future research and clinical applications. Additionally, most studies originate from Chinese contexts, limiting generalizability to diverse populations and healthcare systems.

The theoretical integration of CHAT and CCT, while promising, requires continued empirical validation. Future research should explicitly test CHAT-derived hypotheses about therapeutic mechanisms and developmental processes. Comparative studies with other theoretical frameworks could illuminate CCT’s unique contributions and optimal theoretical foundations.

Quality assessment of existing studies reveals moderate to high risk of bias in some investigations, highlighting the need for more rigorous experimental designs. Future research should prioritize randomized controlled trials with adequate sample sizes, appropriate control conditions, and comprehensive outcome assessments.

## Conclusion

This perspective demonstrates how Cultural-Historical Activity Theory provides a robust framework for understanding Chinese calligraphy therapy’s therapeutic mechanisms and effectiveness. The analysis reveals CCT as a promising culturally mediated intervention that addresses neuropsychiatric symptoms through multiple pathways involving neural efficiency, cultural meaning-making, and social participation. Current evidence supports CCT’s integration into comprehensive mental health approaches, while future research directions promise to expand understanding and optimize clinical applications. The framework’s emphasis on cultural authenticity and developmental processes offers insights for developing culturally responsive mental healthcare that honors traditional practices while meeting contemporary clinical standards.

## Data Availability

The original contributions presented in the study are included in the article/supplementary material. Further inquiries can be directed to the corresponding author.
